# Relationship of mesenteric panniculitis with visceral and subcutaneous adipose tissue

**DOI:** 10.3906/sag-1908-138

**Published:** 2020-02-13

**Authors:** Çiğdem ÖZER GÖKASLAN, Eranıl ASLAN, Emin DEMİREL, Aylin YÜCEL

**Affiliations:** 1 Department of Radiology, Faculty of Medicine, Afyonkarahisar Health Sciences University, Afyon Turkey

**Keywords:** Mesenteric panniculitis, fat tissue, multidetector computed tomography

## Abstract

**Background/aim:**

Mesenteric panniculitis (MP) is an idiopathic benign disease characterized by fat necrosis, chronic inflammation, and fibrosis. The relationship between obesity and chronic low-grade inflammation has been reported. This study investigated the relationship of MP diagnosed using multidetector computed tomography (MDCT) with visceral adipose tissue (VAT) and subcutaneous adipose tissue (SAT) areas.

**Materials and methods:**

We retrospectively enrolled 104 patients with no radiological findings other than MP. Additionally, 76 individuals without any indicative radiological findings were included as controls. VAT and SAT were separately calculated (cm2) using a 3-dimensional workstation. The abdominal circumference was measured (cm).

**Results:**

The mean abdominal circumference was 99.9 ± 7.9 cm, SAT was 195.3 ± 89.1 cm2, and VAT was 203.9 ± 72.8 cm2 in the MP group. The abdominal circumference, VAT, and SAT were significantly higher in the MP group than in the control group (P < 0.001). According to the receiver operating characteristic (ROC) analysis, cut-off level VAT and SAT were 167.5 cm2 (sensitivity 71%, specificity 69%) and 117.5 cm2 (sensitivity 78%, specificity 51 %), respectively.

**Conclusion:**

Increased VAT and SAT were associated with MP, suggesting their role in the etiology of MP.

## 1. Introduction

Mesenteric panniculitis (MP) is an idiopathic benign disease that affects the intestinal mesentery and is characterized by fat necrosis, chronic inflammation, and fibrosis [1,2]. MP has no specific presenting symptoms is usually incidentally diagnosed, and has a prevalence of approximately 0.6% [3].

Although MP usually involves the small bowel mesentery, it can affect the sigmoid mesentery and mesocolon as well [4,5]. However, the specific etiology of this disease remains unknown. The most commonly associated factors are malignancy, granulomatous disease, rheumatologic disease, and previous abdominal trauma or surgery. In addition, studies reported associations of MP with vasculitis, autoimmunity, pancreatitis, ischemic damage, and infection [3,6]. Recently, studies have reported a relationship between obesity and chronic low-grade inflammation and have found higher cytokine levels in patients with obesity [7–11]. However, the relationship of MP with visceral adipose tissue (VAT) and subcutaneous adipose tissue (SAT) is unclear.

Although there are several studies regarding the etiology of MP, as well as those that use body mass index (BMI) in the etiology of MP [12] and its relationship to obesity, there are no studies that investigate the etiology of MP and its relationship to visceral or subcutaneous obesity.

In the present study we investigated the effect of increases in VAT or SAT on the etiology of MP that was incidentally diagnosed using multidetector computed tomography (MDCT). 

## 2. Materials and methods

From our hospital information system, 18,850 patients who underwent abdominal computed tomography (CT) between January 2015 and May 2017 were retrospectively screened to identify those diagnosed with MP. The study was approved by the local ethics committee (approval no.: 2019/162). Among these patients 104 with MP were identified. Furthermore, 76 individuals who underwent abdominal CT for any reason but did not have any indicative radiological findings were included as controls. 

The inclusion criteria were as follows: patients who underwent abdominal CT for abdominal pain, side pain, or any other reason, such as abdominal bloating and/or nausea and the suspicion of renal stones, and who had no CT findings other than incidental MP. The exclusion criteria were as follows: history of abdominopelvic surgery, malignancies causing intra-abdominal inflammation, lymphoproliferative or autoimmune diseases, gastrointestinal diseases (e.g., cholelithiasis, cirrhosis, pancreatitis, peptic ulcer, and retroperitoneal fibrosis), vascular diseases (e.g., mesenteric thrombosis, mesenteric arteriopathy, and abdominal aortic aneurysm), nephrolithiasis, primary abdominal trauma, and intra-abdominal hemorrhage. 

All CT scans were performed using an 80-row detector CT (160 slice) scanner (Aquilion Prime, Toshiba Medical Systems, Nasu, Japan). The CT imaging technique was not standardized due to the variety of different clinical indications. Fourteen patients underwent nonenhanced CT scans (renal stone protocol). Contrast-enhanced images were obtained at the portal venous phase with a start delay of 70 s after each patient received a total of 90–100 mL of nonionic contrast agent and a 30 mL of saline injection at a flow rate of 2–3 mL/s. The CT protocol was as follows: peak kilovoltage 120 kVp, tube current, 150–165 mAs; maximum collimation, 2.5 mm; slice thickness, 2 mm; and rotation time, 0.75 s. 

**Figure 1:**
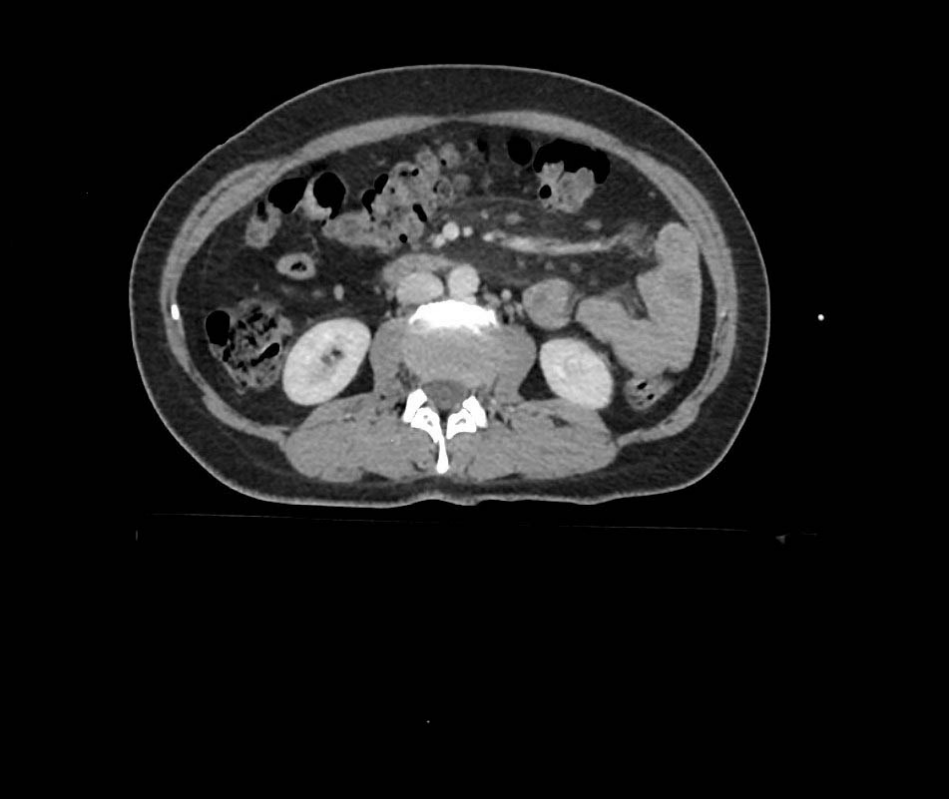
Contrast-enhanced CT scan shows a well-defined mesenteric fatty mass, lymph nodes within the mass, and a surrounding hyperdense pseudocapsule of MP in the left upper quadrant.

A positive CT diagnosis of MP was based on the observation of a well-defined mesenteric fatty mass lesion without infiltration of neighboring structures, an increase in mesenteric fat attenuation, presence of lymph nodes within the mass, presence of a surrounding hyperdense pseudocapsule and limiting mesenteric fat, and presence of a hypodense fatty halo surrounding blood vessels and nodes [13] (Figure 1). All of the study cases satisfied a minimum of 3 of these 5 criteria.

In all patients, fat tissue was assessed from the cross-section of the L2 vertebra using a 3-dimensional workstation (Aquarius 3D Workstation, TeraRecon Inc., San Mateo, CA, USA). The L2 vertebra reference point was selected to standardize the measurement location in all patients. VAT and SAT areas were separately determined (cm2). In addition, abdominal circumference (cm) at the same level was calculated. 

The measurements were performed by 2 radiologists. One of them was a radiologist with 9 years of experience and the other was a 5-year radiology resident. Measurements were repeated twice and averaged.

With this software, VAT and SAT measurements are performed automatically. The software allows manual correction of incorrect drawings. Non-fat tissues detected as fat during measurement were corrected manually. 

**Figure 2:**
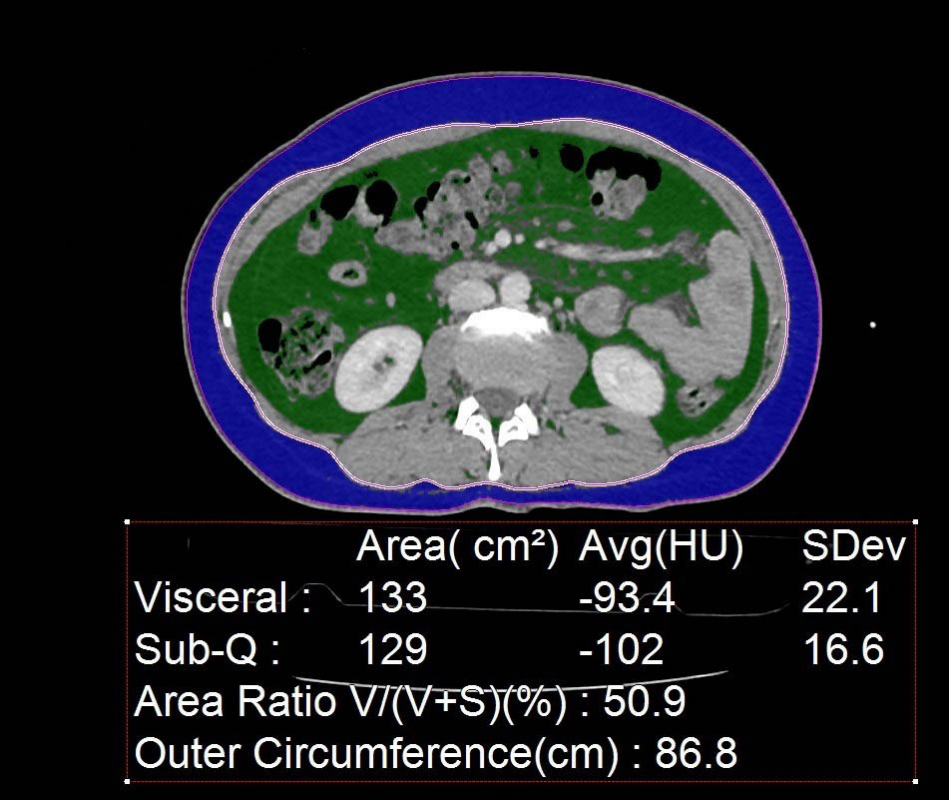
CT image at the L2 vertebral level shows segmentation of VAT (green) and SAT (blue) by the automated tool.

The BMI of each patient was noted. The localization and direction of MP were noted as originating from the small bowel or colon mesenteries (Figure 2). 

All statistical analyses were performed with SPSS statistics software version 24.0 (IBM Corp., Armonk, NY, USA). 

Quantitative data were expressed as mean ± standard deviation (SD), median, and range. Categorical data were expressed as percentage.

The age, BMI, VAT, and SAT areas and abdominal circumference in patients with MP and the controls were examined in terms of their distribution using the Kolmogorov–Smirnov test. Age, BMI, VAT area and abdominal circumference were normally distributed and compared using the t-test. SAT areas were not normally distributed and were compared using the Mann–Whitney U test. The chi-square test was used to compare sex distribution between the groups. 

To determine the diagnostic cut-off for VAT and SAT areas, the receiver operating characteristic (ROC) curve was applied. 

## 3. Results

According to MDCT findings, 104 patients with MP were identified. Of the 104 patients, 50 were male (48.1%) and 54 were female (51.9%), and the mean patient age was 54.7 ± 11.5 years (range, 28–80 years). Among the 76 individuals in the control group, 38 were male (50%) and 38 were female (50%). The mean age of controls was 52 ± 11.3 years (range, 29–75 years), and they were similar to the patients in terms of age and sex (Table 1).

**Table 1:** Demographic characteristics of patients with MP and controls.

	Patient group(n = 104)	Control group(n = 76)
Age (median, mean ± SD) yearsRange (min–max)	55, 54.7 ± 11.5(28–80 years)	52, 52 ± 11.3(29–75 years)
SexFemaleMale	54 (58.7%)50 (56.8%)	38 (41.3%)38 (43.2%)

Among the patients included in this study, MP was diagnosed incidentally, and abdominal CT was performed in 13% of the patients due to abdominal pain.

All cases of MP involved the small bowel mesentery and none originated from the colon mesentery. Among the 104 patients, MP was localized in the right lower quadrant in 2 patients, right upper quadrant in 1patient, left lower quadrant in 7 patients, midline at the para-aortic region in 29 patients, and in the left upper quadrant in 65 patients. 

In the MP group, the mean BMI was 35.8 ± 4.77 kg/m2 (range, 22.5–46.3 kg/m2). A total of 54 patients (51.9%) were classified as obese (BMI >30 kg/m2), and 16 patients (15.4%) presented with morbid obesity (BMI >40 kg/m2). Further, 18 patients (17.3%) presented as overweight (BMI, 25–30 kg/m2), whereas 16 patients (15.4%) in the study population were normal weighted (BMI, 18.5–25 kg/m2). In the control group the mean BMI was 27.6 ± 3.23 kg/m2 (range 19.5–35.7 kg/m2).

In the MP group the mean abdominal circumference was 99.9 ± 7.9 cm, SAT area was 195.3 ± 89.1 cm2, and VAT area was 203.9 ± 72.8 cm2.

In the control group, the mean abdominal circumference was 94.2 ± 12.9 cm, SAT area was 140 ± 106.5 cm2, and VAT area was 135.2 ± 76.1 cm2. 

The BMI, abdominal circumference, VAT, and SAT were significantly higher in the MP group than in the control group (P < 0.001) (Table 2).

**Table 2:** Findings in the MP group and control group.

	Patient	Control	P value
BMI (mean, range) kg/m2	35.8 ± 4.77 (22.5–46.3)	27.6 ± 3.23 (19.5–35.7)	<0.001
Abdominal circumference(cm) Median (mean ± SD)	99.3 (99.9 ± 7.9)	93.0 (94.2 ± 12.9)	<0.001
VAT area (cm2)Median (mean ± SD)	199.0 (203.9 ± 72.8)	131(135.2 ± 76.1)	<0.001
SAT area (cm2)Median (mean ± SD)	183.0 (195.3 ± 89.1)	117 (140 ± 106.5)	<0.001

According to ROC analysis, a cut-off level VAT area was 167.5 cm2 (determined with 71% sensitivity, 69% specificity), and the area under the curve was 0.739 with a 95% confidence interval (CI). 

**Figure 3:**
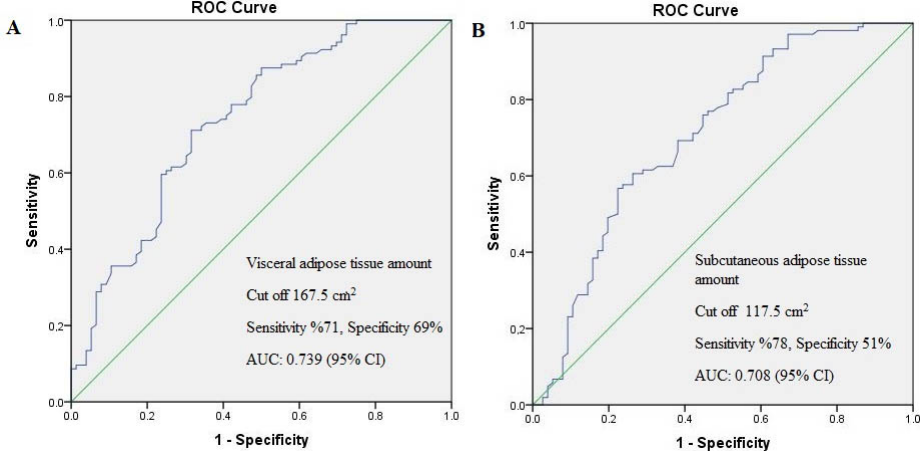
ROC curve of VAT (A) and SAT (B), AUC: Area under the curve, CI: Confidence interval.

A cut-off level SAT area was 117.5 cm2 (determined with 78% sensitivity and 51% specificity), and the area under the curve was 0.708 with a 95% CI (Figure 3).

## 4. Discussion

In the present study we found that the abdominal circumference, VAT and SAT were significantly associated with MP. 

The specific etiology of MP remains unknown. Walled et al. [14] reported that a history of abdominopelvic surgery was present in 49% of patients with MP. Additionally, the authors noted that the prevalence of MP was significantly higher in patients who underwent abdominopelvic surgery than in those who did not undergo abdominopelvic surgery. However, in this previous study there was no mention of BMI or VAT. Furthermore, a history of malignancy was present in 31% of patients with MP. 

Daskalogiannaki et al. [3] reported that 57% of patients with MP underwent previous abdominopelvic surgery, and of these, few patients presented with a mural thrombus in an abdominal aortic aneurysm and ischemic enteritis. The authors suggested that these vascular disorders might have triggered MP. In the present study, patients with a history of surgery and vascular disease were excluded, and the relationship between MP and adipose tissue was investigated.

Unlu et al. [12] assessed 80 patients with MP and reported that the majority of these patients had a high BMI, and 57.5% were obese, 10% were morbidly obese, 17.5% were overweight, and 15% were normal weight. Kaya et al. [15] reported in their study on the diagnosis and treatment of MP that 54.7% of patients with MP had a BMI of >30 kg/m2. The authors suggested that obesity might affect immunity and may be an underlying disorder.

Several programs and tools can calculate visceral and subcutaneous fat by MDCT. Ryckman et al. [16] quantified visceral and subcutaneous abdominal fat using a validated semiautomated software tool. In their study, they investigated whether VAT by CT in asymptomatic adults could predict the possibility of future cardiac events. 

In the literature, no study has investigated the relationship of MP with VAT and SAT using similar CT-based techniques. In the present study we noted a significant relationship between MP and VAT and SAT. Additionally, we estimated a cut-of value for VAT and SAT that may be associated with MP.

In this study, a VAT area greater than 167.5 cm2 and SAT area greater than 117.5 cm2 were significantly associated with MP. 

In previous studies, obese people were compared to people with a normal weight, and the obese people were found to have higher levels of proinflammatory proteins/cytokines, such as acute-phase proteins (C-reactive protein and haptoglobin), interleukin 6, tumor necrosis factor-alpha, adipokines, and neuropeptides (e.g., substance P) [7–11]. All of these proinflammatory factors are produced by adipocytes, as well as macrophages and lymphocytes found in mesenteric adipose tissue. Therefore, a systemic acute-phase response can be triggered with an increase in adipose tissue [8–11]. In addition, adipokines have been shown to be overproduced in the mesenteric adipose tissue of people with inflammatory bowel disease [17–19]. The fact that proinflammatory factors are produced in mesenteric fatty tissue supports the notion of a significant relationship between MP and VAT, as noted in our study. 

The main limitations of our study were that it was retrospective and we had no histological confirmation. Therefore, prospective histological and endocrinological studies are needed to clarify the effects of increased VAT on MP findings.

In conclusion, increased abdominal circumference and VAT and SAT were found to be associated with MP. 

Cut-off levels for VAT and SAT areas of 167.5 cm2 and 117.5 cm2, respectively, were significantly associated with MP. 

## Acknowledgment

The authors thank Professor İsmet Doğan from the department of biostatistics for his valuable input. This research did not receive any specific grant from funding agencies in the public, commercial, or not-for-profit sectors. 

## Informed consent

Informed consent was obtained from all participants of study.
